# Effects of smartphone diaries and personal dosimeters on behavior in a randomized study of methods to document sunlight exposure

**DOI:** 10.1016/j.pmedr.2016.04.002

**Published:** 2016-04-22

**Authors:** Brian Køster, Jens Søndergaard, Jesper Bo Nielsen, Martin Allen, Mette Bjerregaard, Anja Olsen, Joan Bentzen

**Affiliations:** a Department of Prevention and Information, Danish Cancer Society, Strandboulevarden 49, 2100 Copenhagen Ø, Denmark; b Research Unit of General Practice, University of Southern, Denmark; c Electrical and Computer Engineering, University of Canterbury, New Zealand; d Research Centre, Danish Cancer Society, Denmark

**Keywords:** Ultraviolet radiation, Skin cancer, Prevention, Questionnaire, Smartphone, Personal UV dosimetry

## Abstract

Dosimeters and diaries have previously been used to evaluate sun-related behavior and UV exposure in local samples. However, wearing a dosimeter or filling in a diary may cause a behavioral change. The aim of this study was to examine possible confounding factors for a questionnaire validation study. We examined the effects of wearing dosimeters and filling out diaries, measurement period and recall effect on the sun-related behavior in Denmark in 2012.

Our sample included 240 participants eligible by smartphone status and who took a vacation during weeks 26–32 in 2012, randomized by gender, age, education and skin type to six groups: 1) Control + diary, 2) Control, 3) 1-week dosimetry measurement, 4) 1-week dosimetry measurement + diary, 5) 3-week dosimetry measurement and 6) 1-week dosimetry measurement with 4 week delayed questionnaire.

Correlation coefficients between reported outdoor time and registered outdoor time for groups 3–6 were 0.39, 0.45, 0.43 and 0.09, respectively. Group 6 was the only group not significantly correlated. Questionnaire reported outdoor exposure time was shorter in the dosimeter measurement groups (3–6) than in their respective controls.

We showed that using a dosimeter or keeping a diary seems to increase attention towards the behavior examined and therefore may influence this behavior. Receiving the questionnaire with 4 week delay had a significant negative influence on correlation and recall of sunburn. When planning future UV behavior questionnaire validations, we suggest to use a 1-week interval for dosimetry measurements, no diary, and to minimize the time from end of measurement to filling out questionnaires.

## Highlights

• Personal UV dosimeters and diaries were previously applied in studies of UV radiation.
• Effects of using UV dosimeters and diaries on participants' behavior were not previously examined.
• Correlation between objective and subjective measures of outdoor exposure time was shown.
• Their correlation coefficient depends on length of recall (bias).
• The use of dosimeters and diaries increases attention towards the examined behavior.

## 1 Introduction

Exposure to ultraviolet radiation (UVR) is the strongest risk factor for skin cancers of all types, including malignant melanoma (IARC, 2011). The incidence of melanoma (world standardized incidence rate per 100,000) for men and women in Denmark increased from 1.4 and 1.9 in 1949–53 (Engholm et al., 2008) to 20.5 and 25.5 in 2008–12 (Engholm et al., 2012) respectively and is still increasing (Ferlay et al., 2012). Most Danes are fair-skinned and have a high UVR exposure and thus a high risk of skin cancer (Koster et al., 2011a, Koster et al., 2010). Recent surveys (2007–2009) showed that 35% of the population had experienced sunburn in Denmark during the summer (Koster et al., 2010), 29% had used a sunbed (Koster et al., 2009) and 45% had traveled to a sunny destination within the past 12 months (Koster et al., 2011b).

In 2007, a national skin cancer prevention campaign was launched with three primary foci of reducing the UVR exposure of the population; 1) The summer in Denmark, 2) vacationing in sunny countries, and 3) using sunbeds (Koster et al., 2010, Koster et al., 2009, Koster et al., 2011b). The traditional monitoring and evaluation of sun-related behavior is carried out by questionnaires (IARC, 2011). However, these questionnaires are not validated against objective measurements. Monitoring of other health behaviors is validated by objective measurements, e.g. smoking by cotinine measurements, diet by biomarkers, and physical activity by accelerometers and GPS (Kvalvik et al., 2012, McGarty et al., 2014). We currently use annual population-based surveys, where participants have been asked in the beginning of September each year to recall and summarize their behavior in the sun for the past summer or for the past 12 months.

Several kinds of bias could influence this data collection method, and even though this traditional instrument to monitor sun-related behavior is widely used (IARC, 2011), there have been concerns with recall bias (English et al., 1998, Kwok et al., 2009) and selection bias (Boniol et al., 2012). Intensive campaign pressure has increased awareness, but could also lead to social desirability bias (Paulhus, 1991). These considerations led to the initiative of a questionnaire validation project, with the overall aim to optimize the campaign, to more effectively prevent skin cancer. The rationale being that an evaluation, which has been proven significantly associated with a population's actual behavior, is more qualified to be the base of interventions. For example, we may gain new knowledge on the efficiency of specific types of protection behavior, which can be prioritized accordingly.

Correlation between sun-related behavior by a self-reported questionnaire and objective measures of UVR exposure e.g. the use of personal electronic UV dosimeters, was previously shown in local samples. However, wearing a dosimeter is an intervention that could cause a behavioral change. In addition, most studies used diaries to assess the sun-related behavior of their participants (Thieden, 2008, Dwyer et al., 1996, Glanz et al., 2010). Diaries, however, may not be suitable for population-based assessment of UVR exposure. For instance using a diary could influence the participants and induce a change of behavior. Effects of using a diary or wearing a dosimeter were to our knowledge not previously described. Glanz et al. made an indirect questionnaire validation of outdoor exposure, by comparing first dosimeters and diaries and then diaries with questionnaires in a study of children and lifeguards (Glanz et al., 2010). Recently, a small study validated a brief questionnaire of sun exposure directly against objective measures of UVR exposure including UVR dosimeters (Cargill et al., 2012). Cargill et al. reported a significant association between outdoor times reported in a questionnaire and registered on a UV dosimeter.

The overall aim of this study was to develop the best conditions for a questionnaire validation study. Here we describe possible intervention effects; feasibility was previously described (Koster et al., 2015). We examined smartphones as a new media for monitoring sun-related behavior and we examined measures of outdoor time from questionnaires and actual outdoor time exposure registered by personal electronic UV dosimeters. We tested effects of wearing dosimeters in studies of sun-related behavior including intervention effects of dosimeters and diaries, measurement period and recall effects.

## 2 Method

### 2.1 Study design and participants

Participants were recruited in May 2012 through the Facebook site and the newsletter of the Danish Cancer Society, and were eligible to this study, if they were living in Denmark and vacationing in Denmark during the week 26, 28, 30 or 32 (late June to mid-August). Participants were randomly assigned to a dosimeter group (which were instructed to wear a dosimeter, complete a short daily sun diary and a questionnaire at the end of the measurement period) or control group (which received the diary and questionnaire, but not a UV dosimeter) by vacation week. Participants for groups using a diary were recruited among regular smartphone users and received their diary to fill in on the smartphone. Participants for 3 week measurement were restricted to persons volunteering for and having 3 weeks of vacation. The study sample was randomized by gender, age (15–34, 35–54, 55 +), education (3 levels) and skin type into six groups as shown in Table 1: 1) Control + diary, 2) Control, 3) 1 week dosimetry measurement, 4) 1-week dosimetry measurement + diary, 5) 3 week dosimetry measurement and 6) 1 week dosimetry measurement with 4 week delayed questionnaire. The randomization procedure aimed to produce six equal groups and to achieve the best representation i.e. groups least represented in recruitment e.g. males and young people were randomized first and then subsequent groups in surplus were randomized. BK designed the randomization and MB assigned the participants to interventions. Participants in the dosimetry groups were sent a dosimeter and a prepaid envelope. Groups 1 and 4 received a diary each of the seven days. Groups 1–5 subsequently received a longer questionnaire one week after the measurement period, whereas group 6 received it with 4 week delay. Group sizes varied according to length of vacation, smartphone use and balance of background variables. The detailed description of recruitment, study flow and use of dosimeters was previously described (Koster et al., 2015). The participants' use of the dosimeter was previously shown to be less than 100% of the time and measurements were adjusted according to the time period the dosimeter was actually carried (Koster et al., 2015).

### 2.2 Diary

The participants were sent a text message with a unique link for a survey platform (www.surveyxact.dk) to a diary each of the seven days at about 7 pm. The diary included “Did you wear the dosimeter today?”, “How many hours were you outdoor between 7 and 11 am; 11 am and 3 pm; 3 and 7 pm?”, “Did your skin get reddish today?”, and “How was the weather today?”.

### 2.3 Questionnaire

The participants were sent a longer questionnaire on their outdoor behavior in the measurement period, sun protection, sunburn and on the use of the dosimeter e.g. “How many hours were you outdoor between 11 am and 3 pm?”, “How many days did you wear the UV dosimeter?”, “How was the weather during the measurement week?”, “How much of the time spend outdoors between 7 am and 7 pm did you wear the dosimeter?”, “When did you wear it”, “When you wore the dosimeter, were you attentive towards the dosimeter?”, and “When you wore the dosimeter, did other people notice or comment on the dosimeter?”. The questionnaire also included questions on knowledge and attitude towards sun-related behavior. The questionnaire was developed from the annual monitoring questionnaires of the Danish Cancer Society (www.skrunedforsolen.dk), and retrieved material from other researchers (Branstrom et al., 2010, Diffey and Norridge, 2009) supplemented with new not validated questions. A translated version of the questionnaire can be found on the project page www.mituv.dk and as Supplementary material.

### 2.4 Statistics

Data on sun-related behavior were dichotomized and tested with background variables for statistical significance by χ^2^ statistics and logistic regression. Crude and adjusted odds ratios (ORs) and 95% confidence intervals (CIs) were calculated. Correlation between responses from questionnaire and dosimeters was examined using parametric and non-parametric statistics. For all tests, p values < 0.05 were considered statistically significant. We used SAS version 9.3 (SAS Institute, Cary, North Carolina, USA) for the analyses.

The project was sent to The National Committee on Health Research Ethics who decided that their approval was not necessary. Danish Data Protection Agency gave approval number 2012-41-0100.

## 3 Results

In total, 585 persons volunteered for the project. Of those 526 were successfully contacted by phone and 306 persons were included to the study by measurement week and number of available dosimeters. Sixty-six persons either did not have a successful dosimeter measurement or did not complete the questionnaire and the final sample for analysis included 240 persons. Table 1 shows the final distribution of background variables and assigned intervention by groups. The proportion of men was significantly higher in group 5 (3 weeks). For age-group, skin type, region and education there were no statistically significant differences; however, the proportion of persons aged 55 or above was slightly lower in groups 1 and 4 which also had higher proportions of persons from the capital region.

### 3.1 Correlation of registered and reported exposure

Table 2 shows reported outdoor time from the final questionnaire, time with positive registered exposure, exposure in standard erythema doses (SED) and correlation coefficients between reported outdoor time and registered outdoor time for groups 3–6. Questionnaire reported outdoor exposure time was shorter in groups with dosimetry measurement (3–6), however not statistically significantly different from the relevant control groups (1–2). Control + diary (group 1) was higher than 1-week dosimetry measurement + diary (group 4) and control (group 2) higher than 1-week dosimetry measurement (group 3). Group 2, which did not receive any intervention, reported the longest outdoor exposure time, while group 4 receiving both a diary and dosimeter reported the shortest outdoor exposure time; these two groups were significantly different. Regarding the actual exposure measured by the UV dosimeter, group 3 received a higher daily dose than group 4 in accordance with self-reported results. Group 6 had the lowest exposure and a relatively low exposure in comparison with self-reported results. There were no between-group differences for the objectively measured estimates, neither registered time or dose. All measurement groups except group 6 demonstrated statistically significant correlations between reported exposure and registered exposure.

### 3.2 Intervention effects

Table 3 shows differences between sunburn, behavior towards two different types of sun protection and intention to tan between groups. The results are based on questions on the frequency of sunscreen use and use of shade and number of sunbathing sessions. Groups 1–6 were combined to examine each of the effects of using a dosimeter, receiving a diary, duration of measurement and delay of a questionnaire. There were no differences for any of the behaviors between participants using a dosimeter or diary. Participants that were measured for 3 weeks sunbathed more often; however this was an expected effect as respondents were summarizing over a longer period and this question was not proportionate. Persons receiving the questionnaire with a 4 week delay reported less sunburn and less sunbathing.

Table 4 shows logistic regression analysis of intervention effects on sun-related behavior in two models including gender, age, education, skin type and ambient UV-radiation i.e. the local weather. In addition, they also included respectively the objective and subjective measures of the actual exposure. Participants that use dosimeters reported sunburn more often when analyzed with the subjective measure. Persons receiving a delayed questionnaire less frequently reported sunburn as analyzed with both measures.

The 240 participants' assessment of how wearing a dosimeter influenced them showed that 67% said that they were attentive towards the dosimeter, but that they did not behave differently; 16% said that they were attentive towards the dosimeter and that they were more careful in the sun and 16% said that they did not pay any attention towards the dosimeter at all. Also when asked about other people's reaction towards the dosimeter, 28% said that a lot of people noticed it, 49% said that some noticed it and 23% said that no one noticed it.

The two different analyses applied in Table 4, which either used the registered or reported measure, mostly yielded results with similar directions; however there were some differences.

## 4 Discussion

This study showed significant effects of interventions in studies of exposure to ultraviolet radiation. Self-reported outdoor time was lower with dosimeter and diary combined than among control groups. Receiving a delayed questionnaire resulted in a poor correlation between reported and registered outdoor times. In addition, our results indicate underreporting of sunburn and sunbathing among persons, who received the questionnaire with a delay. More persons reported sunburn when wearing a dosimeter. We did not find significant effects on sun protective behavior.

### 4.1 Strength and limitations

The size of our final sample was modest and some effects may exist even though we were not able to show these and should be tested in a larger sample. Unfortunately, there was a larger decline in the group of delayed questionnaires, which resulted in a smaller group. The decline was caused partly by dosimeter flaws and partly due to resignation towards answering the questionnaire. The group with delayed questionnaires could be different from the other groups, as there was a tendency towards a lower UV dose; however there were no significant differences. The randomization procedure was replaced by random matching as described for e.g. persons having 3 weeks of vacation; however, the only difference between groups was a higher percentage of men in this group. It is assumed not to have introduced any bias, as the final analysis was adjusted for gender.

The participants were instructed very carefully to make sure the dosimeter was used correctly, i.e. not covered by sleeves, worn all the time etc. and self-reported compliance was high; however we cannot know for sure if instructions were followed strictly and if not this could cause some disagreement between self-reported measures and objectively measured exposure.

The differences between objectively and subjectively measured times were previously shown to be partly explained by the fact that dosimeters worn on wrists measure about half of the UV-exposure as compared to dosimeters worn on the head (Thieden et al., 2000) in combination with an overestimation in questionnaires as compared to e.g. diaries (Koster et al., 2015). As the overestimation was proportional to the exposure, we assume that it did not influence our results.

### 4.2 Effects of smartphone diaries and dosimeters

In behavioral studies, results could be biased by a given intervention. Wearing a device monitoring participants’ behavior could be described as Hawthorne effects i.e. people behave differently when given extra attention (Best & Neuhauser, 2006). More than 80% of the participants were attentive towards the dosimeter and 16% admitted to behaving differently. It must be assumed that these numbers could be a concern in behavioral studies. The differences in reported outdoor time between groups wearing dosimeters and receiving diaries vs. control groups, could be caused by both more attention towards correct reporting and change of behavior. Persons receiving diaries only registered slightly less outdoor time/UV-dose and at the same time persons wearing a dosimeter reported more sunburns. This could imply that both effects are related to increasing attention towards the reporting. Persons wearing a dosimeter know that they are being measured and thus are more attentive towards the immediate effects of too much UV-radiation i.e. sunburn (Best and Neuhauser, 2006, Strub, 1989). The use of a diary or a dosimeter could make you more aware of sun-related behavior, but we did not show any significant effects. This could mean that the type of effect seen is more like a panopticon (Strub, 1989) effect than a Hawthorne effect (Best & Neuhauser, 2006), i.e. it is not the attention alone, but the transparency of surveillance that possibly could have an effect. In addition, as we do not have an actual measure of the exposure we cannot say for sure that there was an effect of dosimeters at all. Using diaries are on one side an intervention, but on the other side a slightly more accurate measure than questionnaires only (Koster et al., 2015).

### 4.3 Duration and delay

The length of an intervention and the time from intervention to evaluation are both factors prone to introduce bias especially the latter. The length needs to be long enough to obtain a true picture of the behavior, but not too long to avoid bias due to decreased compliance and recall bias. We did not find any unexpected effects from different intervention durations implying that durations of 1–3 weeks would be applicable for a validation study. We showed that the time from intervention to evaluation is absolutely crucial as the correlation of objectively and subjectively measured exposure did almost not exist with a 4 week delay. In addition, recall bias seems to have caused underreporting of sunburn despite accordance with results from objectively measured exposure. There were no effects on recall effects on reported behavior though. The lack of effects on protection from the sun could be caused by the fact, that how you usually protect yourself from the sun is a long lasting habit, which does not change over time (Idorn et al., 2014). The results with opposite directionality in Table 4 when using registered versus reported results in the analysis underline the importance and need of validated questionnaires. Together with the results that show that a recall period of four to six weeks yields subjective estimates in little or no agreement with objective measurements, the set-up of traditional questionnaires of sun-related behavior may need to be re-thought.

When planning future UV behavior questionnaire validations we suggest to use a 1-week interval for dosimetry measurements, no diary, and to minimize the time from end of measurement to filling out questionnaires.

## 5 Conclusion

We conclude that monitoring of sun-related behavior using diaries and dosimeters seems to be influenced by the measurement methods or may increase attention towards the behavior examined; however, certain measures can be taken to optimize measurements. We also conclude, that to be able to conduct a questionnaire validation of the actual behavior without influencing the outcome, it is thus important to keep interventions at a minimum in the measurement period. It is a balance between (not) influencing behavior and measurement precision. Finally, we conclude that, when using questionnaires to evaluate exposure and behavior, reducing time from the period monitored to evaluation is of utmost importance.

## Funding

This study was supported by TrygFonden and The Danish Board of Research and Innovation.

## Conflicts of interest

None declared.

## Transparency document

Transparency document. Image 1

## Figures and Tables

**Table 1:** Distribution of background variables by groups and distribution of group interventions in Denmark in 2012.

Characteristic (%) Total (n = 240)	Total	Group 1 (n = 33)	Group 2 (n = 41)	Group 3 (n = 66)	Group 4 (n = 41)	Group 5 (n = 35)	Group 6 (n = 24)
Used diary	Yes	No	No	Yes	No	No
Used dosimeter	No	No	Yes	Yes	Yes	Yes
Weeks of participation	1	1	1	1	3	1
Questionnaire recall time	< 2 weeks	< 2 weeks	< 2 weeks	< 2 weeks	< 2 weeks	4–6 weeks
Gender	p = 0.036						
Male	20	12	17	21	15	40	13
Female	80	88	83	79	85	60	88
Age group	p = 0.325						
15–24	15	12	12	11	20	17	21
25–34	17	15	10	11	24	26	21
35–44	16	27	15	15	15	14	8
45–54	22	21	24	23	29	20	8
55–64	19	18	24	27	7	9	25
65 +	12	6	15	14	5	14	17
Skin type	p = 0.393						
I	15	12	10	12	20	11	29
II	58	70	66	55	49	63	46
III/IV	28	18	24	33	32	26	25
Region	p = 0.544						
Capital	33	45	29	24	49	29	21
Zealand	15	9	15	20	10	11	25
Northern Jutland	10	9	17	8	7	11	8
Central Jutland	20	12	20	26	15	26	21
Southern Denmark	22	24	20	23	20	23	25
Education	p = 0.056						
Primary school	10	6	17	6	5	17	13
Secondary school	14	0	12	8	24	20	25
Vocational	13	12	10	14	12	23	4
Higher education (< 2 years)	15	18	17	21	5	9	13
Higher education (2–4½ years)	38	42	34	42	44	23	38
Higher education (> 4½ years)	11	21	10	9	10	9	8

**Table 2:** Reported and registered outdoor time and exposure and correlation between reported questionnaire estimate and registered UV-measurement in daily outdoor time in Denmark in 2012.

Group^a^	n	Minutes (questionnaire)	Minutes (reg)	SED/day	Corr. coef.
1)	33	280 (229–331)	n.a.	n.a.	n.a.
2)	41	317 (273–361)	n.a.	n.a.	n.a.
3)	66	296 (254–338)	62 (34–99)	1.1 (0.5–1.8)	0.39, p = 0.001
4)	41	230 (195–266)	53 (23–92)	0.8 (0.3–1.5)	0.45, p = 0.003
5)	35	288 (231–345)	41 (19–60)	0.7 (0.4–1.1)	0.43, p = 0.011
6)	24	269 (207–330)	26 (13–51)	0.4 (0.2–0.8)	0.09, p = 0.676

Reported exposure is mean (95CI) and registered exposure is median (q1, q3). The objective measured results were adjusted for number of days not wearing the dosimeter in the measurement period.

a 1) Control + diary, 2) Control, 3) 1 week dosimetry measurement, 4) 1-week dosimetry measurement + diary, 5) 3 week dosimetry measurement and 6) 1 week dosimetry measurement with 4 week delayed questionnaire.

**Table 3:** Distribution of sun-related behavior in percentage by examined intervention effects in Denmark in 2012.

Characteristic (%) Total (n = 240)	Dosimeter			Diary			Duration			Recall period		
Yes n = 166	No n = 74		Yes n = 74	No n = 166		3-weeks n = 35	1-week n = 205		4–6 weeks n = 24	0–2 weeks n = 216	
SunburnedOverall (dichotomized)	34	24	p = 0.145	30	31	p = 0.805	43	29	p = 0.071	12	34	**p** **=** **0.026**
Use of sunscreen > SPF15Often/always (60–100% of the time)	31	39	p = 0.199	32	34	p = 0.843	34	33	p = 0.897	25	34	p = 0.361
Use of shade between 12 & 15Often/always (60–100% of the time)	15	19	p = 0.454	18	16	p = 0.712	23	15	p = 0.252	21	16	p = 0.521
SunbathedOverall (dichotomized)	50	53	p = 0.667	45	51	p = 0.393	71	46	**p** **=** **0.005**	29	52	**p** **=** **0.037**

Distribution of sunburn, protection and risk behavior between groups. Values are percent and p-values are given for Chi-Square of observed vs. expected numbers. Statistically significant results are shown in bold.

**Table 4:** Logistic regression analysis of intervention effects on sun related behavior adjusted for objective or subjective measure of exposure in Denmark in 2012.

Characteristic Total (n = 240)	Models with objective estimate			Models with subjective estimate			
p-Value OR (95CI)	Text-message diary (n = 106)	Duration (n = 101)	Recall period (n = 90)	Dosimeter (n = 180)	Text-message diary (n = 180)	Duration (n = 101)	Recall period (n = 90)
SunburnedOverall (dichotomized)	p = 0.281	p = 0.997	p = 0.078	**p** **=** **0.029**	p = 0.994	p = 0.812	p = 0.090
0.45 (0.10–1.93)	1.00 (0.29–3.86)	0.19 (0.03–1.02)	**2.37 (1.11–5.26)**	1.00 (0.39–2.55)	0.87 (0.25–2.84)	0.21 (0.27–1.12)
Use of sunscreen SPF15Often/always (60–100% of the time)	p = 0.116	p = 0.787	p = 0.787	p = 0.463	p = 0.494	p = 0.732	p = 0.759
0.32 (0.07–1.26)	1.23 (0.26–5.78)	1.23 (0.26–5.78)	0.76 (0.36–1.60)	0.76 (0.34–1.68)	1.30 (0.28–6.13)	0.84 (0.25–2.56)
Use of shade (12–15)Often/Always (60–100% of the time)	p = 0.622	p = 0.224	p = 0.379	p = 0.137	p = 0.589	p = 0.124	p = 0.323
1.38 (0.38–5.01)	2.15 (0.63–7.68)	1.93 (0.43–8.20)	0.51 (0.20–1.24)	1.27 (0.53–3.05)	2.65 (0.77–9.55)	2.06 (0.47–8.68)
SunbathedOverall (dichotomized)	p = 0.350	**p** **=** **0.002**	p = 0.843	p = 0.801	p = 0.592	**p** **=** **0.008**	p = 0.647
0.54 (0.14–1.95)	**5.64 (1.87–17.07)**	1.22 (0.16–9.48)	0.91 (0.43–1.91)	1.24 (0.57–2.79)	**4.21 (1.46–12.16)**	0.70 (0.14–3.25)

The models included gender, age education, region, skin type, weather in measurement period interventions, and respective measure of exposure. Statistically significant results are shown in bold.

## Appendix A Supplementary data

Supplementary material. Image 2
